# Nasopharyngeal Colonization and Invasive Disease Are Enhanced by the Cell Wall Hydrolases LytB and LytC of *Streptococcus pneumoniae*

**DOI:** 10.1371/journal.pone.0023626

**Published:** 2011-08-23

**Authors:** Elisa Ramos-Sevillano, Miriam Moscoso, Pedro García, Ernesto García, Jose Yuste

**Affiliations:** Centro de Investigaciones Biológicas, Consejo Superior de Investigaciones Científicas, and CIBER de Enfermedades Respiratorias (CIBERES), Madrid, Spain; University, Complutense, Spain

## Abstract

**Background:**

*Streptococcus pneumoniae* is a common colonizer of the human nasopharynx and one of the major pathogens causing invasive disease worldwide. Dissection of the molecular pathways responsible for colonization, invasion, and evasion of the immune system will provide new targets for antimicrobial or vaccine therapies for this common pathogen.

**Methodology/Principal Findings:**

We have constructed mutants lacking the pneumococcal cell wall hydrolases (CWHs) LytB and LytC to investigate the role of these proteins in different phases of the pneumococcal pathogenesis. Our results show that LytB and LytC are involved in the attachment of *S. pneumoniae* to human nasopharyngeal cells both in vitro and in vivo. The interaction of both proteins with phagocytic cells demonstrated that LytB and LytC act in concert avoiding pneumococcal phagocytosis mediated by neutrophils and alveolar macrophages. Furthermore, C3b deposition was increased on the *lytC* mutant confirming that LytC is involved in complement evasion. As a result, the *lytC* mutant showed a reduced ability to successfully cause pneumococcal pneumonia and sepsis. Bacterial mutants lacking both LytB and LytC showed a dramatically impaired attachment to nasopharyngeal cells as well as a marked degree of attenuation in a mouse model of colonization. In addition, C3b deposition and phagocytosis was more efficient for the double *lytB lytC* mutant and its virulence was greatly impaired in both systemic and pulmonary models of infection.

**Conclusions/Significance:**

This study confirms that the CWHs LytB and LytC of *S. pneumoniae* are essential virulence factors involved in the colonization of the nasopharynx and in the progress of invasive disease by avoiding host immunity.

## Introduction

*Streptococcus pneumoniae*, the pneumococcus, is a major cause of bacterial sepsis and the most common etiologic agent in acute otitis media, community-acquired pneumonia as well as non-epidemic bacterial meningitis [1], [2]. Pneumococcal disease is preceded by colonization, which is particularly common in children, with more than one serotype frequently colonizing the nasopharynx of the same individual at the same time [1]. Direct bacterial translocation from the nasopharynx to the bloodstream, generally known as occult bacteremia, is a well-recognized complication of pneumococcal carriage, particularly in early childhood [3]. Pneumococcal colonization involves binding of the bacterium to cell-surface carbohydrates such as N-acetyl-glycosamine on the respiratory epithelium and this process is mediated by cell-wall-associated surface proteins [1]. Cell wall hydrolases (CWHs) are surface enzymes that cleave specific covalent bonds of the cell wall and eventually, cause the lysis and death of the bacteria [4]. Among these proteins, LytB is a CWH located at sites closely related to the polar ends of the cell. This enzyme has N-acetylglucosaminidase activity and plays an essential role in daughter cell separation [5], [6]. Moreover, it has been suggested that LytB, regardless of being polymorphic [7], might be a promising target for the development of a universal pneumococcal vaccine because an anti-LytB antiserum significantly protected mice from a lethal challenge with different pneumococcal strains [8]. Other well-characterized CWHs include the LytC lysozyme that, in contrast to LytB, has a general cell-surface distribution [9]. LytC is a protein that behaves as an autolysin at 30°C and the fact that it has its maximum enzymatic activity at this temperature suggests that it might be more crucial in the upper respiratory tract [10]. LytC is one of the major bacterial components that enable *S. pneumoniae* to lyse non-competent pneumococci (fratricide) [11]. Indeed, fratricide has been proposed as a mechanism of predation that contributes to virulence by regulating the release of several virulence factors [11].

Although much work has been done under in vitro conditions, whether CWHs are important virulence factors involved in direct interaction with the host surfaces or in the establishment of the pneumococcal pathogenesis has not been clearly defined. As a common colonizer of the upper respiratory tract, *S. pneumoniae* has developed an arsenal of components that are of great importance for biofilm formation and efficient colonization of the nasopharynx which is the first step of pneumococcal virulence [12]. Inactivation of LytB and LytC has been shown to hinder biofilm formation whereas simultaneous disruption of both CWHs markedly reduced biofilm establishment, which may suggest that both enzymes are important pneumococcal components with additive or synergistic effects upon bacterial adhesion [13]. In this sense, using single *lytB* and *lytC* mutants on an unencapsulated type 4 strain, it has been shown that loss of LytC but not LytB showed a moderate impaired attachment to nasopharyngeal cells at 30°C but not a 37°C whereas no attenuation was found in pneumococcal sepsis for both mutations [14]. Besides, as the most common cause of community acquired pneumonia, the ability of *S. pneumoniae* to invade the pulmonary compartment and escape from the immune system is a key event in the pathogenesis of pneumococcal pneumonia and invasive disease. Therefore, the characterization of virulence factors involved in pathogenesis is necessary to understand the molecular basis of pneumococcal disease and allow identification of new targets for the prevention and treatment of *S. pneumoniae* infection.

In this study, we have constructed isogenic mutants lacking LytB and/or LytC to analyze their role in the engagement and persistence of *S. pneumoniae* in the upper respiratory tract. We have also investigated the role of these proteins in evasion of complement immunity and phagocytosis. Finally, we have explored the implication of LytB and LytC in the establishment of pneumococcal pneumonia and bacterial dissemination throughout the systemic circulation.

## Materials and Methods

### Ethics Statement

The part of the study including serum sampling from healthy volunteer controls as a source of complement was approved by the Centro de Investigaciones Biológicas (CIB) Research Ethics Committee and was obtained following institutional guidelines. Animals were bred at our Institution's animal facilities whereas the scientific procedures were performed at Fundación Jiménez Díaz (FJD) following institutional guidelines for animal use and care. Infection experiments conformed to the Spanish government legislation (RD 1201/2005) and European Community regulations (86/609/EEC). The Animal Care and Use Committees of both Institutions approved all the experiments involving animals in this study (Approval Reference: CIB-FJD 06010017).

### Bacterial strains and growth conditions

The *S. pneumoniae* clinical isolates used in the present study were TIGR4 (serotype 4) [15] and D39 (serotype 2) [16]. Isogenic *lytB* or *lytC* mutants of TIGR4 and D39 strains were constructed by transformation with DNA prepared from mutants previously characterized in our laboratory [5], [13]. Kanamycin (250 µg ml^−1^) and tetracycline (0.5 µg ml^−1^) were added to blood agar plates when required. The *lytB lytC* double mutants were constructed by transforming *lytB* mutants with DNA obtained from a *lytC* strain. The accuracy of the mutations was confirmed by PCR using specific primers. *S. pneumoniae* strains were incubated at 37°C on blood agar plates (with or without antibiotics) in a 5% CO_2_ atmosphere, or in Todd-Hewitt broth supplemented with 0.5% yeast extract, to an optical density at 550 nm of 0.5, corresponding to *ca*. 10^8^ CFU ml^−1^, and stored at –70°C in 10% glycerol as single-use aliquots.

### In vitro studies

#### a) Attachment to nasopharyngeal cells

Monolayers of human nasopharyngeal Detroit-562 (D562) cells (CCL138; ATCC) were cultured to 90–95% confluence in tissue culture flasks or in plates containing RPMI 1640 supplemented with 10% heat-inactivated fetal calf serum, 1% glutamine, and 1 mM sodium pyruvate [17]. Tissue culture 24-well plates containing 10^5^ cells per well were infected in triplicate with the pneumococcal isolates at a ratio of 10 bacteria:1 cell and incubated at 30°C or 37°C for 1 h. Afterwards, infected plates were washed three times with phosphate-buffered saline (PBS; pH 7.3) and adherent bacteria were lifted off by treatment firstly with 200 µl per well of a solution containing 0.25% trypsin-1 mM EDTA and then with 200 µl per well of 0.025% Triton X-100 (in PBS) as previously described [17]. To determine the proportion of bacteria recovered from infected cells, serial dilutions were plated and counted. Experiments were repeated three times and results were expressed as the proportion of bacteria recovered from D562 cells infected with the different mutants in comparison to the control groups of cells infected with the D39 and TIGR4 wild-type strains.

#### b) C3b binding to *S. pneumoniae*

Serum samples from five healthy male volunteer controls (median age of 40 years) were obtained according to institutional guidelines and stored as single-use aliquots at –70°C to use as a source of complement. C3b deposition on the *S. pneumoniae* surface was analyzed using a flow cytometry assay [18], [19]. Briefly, C3b deposition was investigated by incubating 10^7^ CFU of *S. pneumoniae* with 10 µl of pooled human serum (diluted to 20% in PBS) for 20 minutes at 30°C or 37°C. After two washes in PBS-Tween 20 (0.01%), C3b bound to the different strains was labeled with 50 µl of a 1/500 dilution of fluorescein isothiocyanate-conjugated polyclonal goat anti-human C3b antibody (ICN). The detection of C3b binding was performed using a FACS Calibur flow cytometer (BD Biosciences) with gating based on the analysis of at least 25,000 bacteria [18]. Experiments were repeated three times and the results were expressed as the proportion of C3b deposition on the surface of the different mutants compared to the C3b deposition on the D39 wild-type strain. Bacteria incubated with PBS instead of serum were included as a negative control [18], [19]. Additional experiments of C3b deposition were performed in the presence or absence of 2 µg of the purified proteins LytB and/or LytC to assess restoration of C3b levels.

#### c) Phagocytosis of *S. pneumoniae*

c1- By mice alveolar macrophages. Experiments testing murine alveolar macrophage phagocytosis were performed as previously described [20]. Briefly, monolayers of MH-S cells (CRL-2019; ATCC) were grown in RPMI 1640 tissue culture medium supplemented with 10% heat-inactivated fetal calf serum and HEPES (10 mM). Cells seeded in 24-well plates containing 7×10^5^ cells per well were infected in triplicate with 50 µl of a suspension of the pneumococcal isolates at a ratio of 50 bacteria:1 cell and incubated at 37°C. For adhesion assays, cells were infected for 1 h, washed five times with PBS and lysed with 300 µl of a solution containing 0.025% saponin-PBS for 10 minutes at room temperature. Viable bacteria recovered from infected cells were obtained by plating serial dilutions on blood agar plates. For phagocytosis assays, cells previously infected with the different strains for 1 h were washed five times with PBS and incubated for an additional hour in tissue culture medium containing penicillin (10 µg/ml) and gentamicin (200 µg ml^−1^) to kill extracellular bacteria. Bacterial counts were obtained from cells washed three times with PBS and lysed as explained above. Experiments were repeated three times and results were expressed as CFU/ml of bacteria recovered from MH-S cells infected with the different mutants in comparison to cells infected with the D39 wild-type strain as a control.

c2- By human neutrophils. Experiments investigating human neutrophil phagocytosis were performed using a flow cytometry opsonophagocytic assay including *S. pneumoniae* labeled with 5, 6-carboxyfluorescein succinimidyl ester (Molecular Probes) and HL-60 cells (CCL-240; ATCC) differentiated to granulocytes [21], [22]. This study was performed using the D39 wild type strain and isogenic single *lytB*, *lytC* and the double *lytB lytC* mutant strains. Differentiation into granulocytes was confirmed before the assays using a monoclonal antibody to CD11b (kindly supplied by Prof. C. Bernabeu, CIB-CSIC) which is a marker of granulocytic differentiation [23]. A minimum of 6,000 cells were analyzed using a Cytomics FC500 Beckman Coulter flow cytometer equipped with a 488 nm Ar-ion laser. Experiments were repeated three times and bacteria incubated in PBS instead of pooled human serum (20% diluted in PBS) were used as a negative control. Results were expressed as a fluorescence index defined as the proportion of positive cells for fluorescent bacteria multiplied by the geometric mean of fluorescence intensity which correlates with the amount of bacteria phagocytosed per cell [21], [24]. Additional experiments of phagocytosis by human neutrophils were performed in the presence or absence of 2 µg of the purified proteins LytB and/or LytC to assess prevention of phagocytosis by the CWHs LytB and LytC of *S. pneumoniae*.

### In vivo studies

#### a) Colonization mice model

To investigate the ability of the wild-type strains to induce a prolonged colonization of mice, groups of five C57BL/6 mice (8 to 16 weeks old) under anesthesia with isofluorane were inoculated by the intranasal route with 10 µl of a suspension containing 10^9^ CFU/ml of each of the wild-type strains (TIGR4 and D39). Animals were sacrificed daily during a period of 7 days and bacterial counts were obtained from the nasopharyngeal lavage fluid. For the studies analyzing nasopharyngeal colonization of the wild-type strains (used as controls) vs. the single and double mutant strains, groups of at least five C57BL/6 mice under anesthesia with isofluorane were inoculated intranasally with 10^9^ CFU/ml (in a volume of 10 µl containing 10^7^ CFU) of each strain in separate groups of mice [25], [26]. At 24 h and 120 h after challenge, a lethal dose of pentobarbital was administered. Nasopharyngeal lavage fluid was collected, diluted and plated for determination of viable bacteria. Experiments were repeated twice using 5 mice in each group. Results were expressed as Log CFU/ml of bacteria recovered from the nasopharyngeal lavage fluid.

#### b) Pneumonia and sepsis mice model

For experiments investigating the role of the different CWHs in the establishment of pneumococcal pneumonia and sepsis, inoculation and sample collection were performed in groups of 5 CD-1 mice (8 to 16 weeks old) as previously described [18]. Mixed infection experiments in a 1∶1 ratio, were used to calculate the competitive index (CI) defined as the ratio of the test strain (single or double mutant) compared to the control strain (wild-type or a single mutant strain, respectively) recovered from mice divided by the ratio of the test strain to the control strain in the inoculum [18], [27]. A CI of <1 indicates that the test strain is attenuated in virulence compared to the control strain. The lower the CI the more attenuated the strain. For the sepsis model of infection, mice were inoculated by the intraperitoneal route with a total of 5×10^5^ CFU/ml (in a challenge suspension of 200 µl containing 5×10^4^ CFU of each strain as a mixed infection in a 1∶1 ratio) and the CI was calculated for bacteria recovered from the spleen after 24 h. For the pneumonia model, mice under anesthesia with isofluorane were infected by the intranasal route with a total of 4×10^8^ CFU/ml (in a volume of 50 µl containing 1×10^7^ CFU of each strain as a mixed infection in a 1∶1 ratio) and the CIs were calculated from bacteria recovered from bronchoalveolar lavage fluid (BALF), lung and blood after 24 h. CI experiments were repeated twice using 5 mice in each group and were performed using D39 wild-type strain and isogenic *lytB*, *lytC* and the double *lytB lytC* mutant strains.

### Statistical analyses

Data are representative of results obtained from repeated independent experiments, and each data point represents the mean and standard deviations (SD) for 3 to 5 replicates. Statistical analysis was performed by using two-tailed Student's *t* test (for two groups), whereas analysis of variance (ANOVA) was chosen for multiple comparisons. GraphPad InStat version 3.0 (GraphPad Software, San Diego, CA) was used for statistical analysis.

## Results

### LytB and LytC are important surface proteins involved in the attachment of *S. pneumoniae* to human nasopharyngeal cells

**Figure 1:**
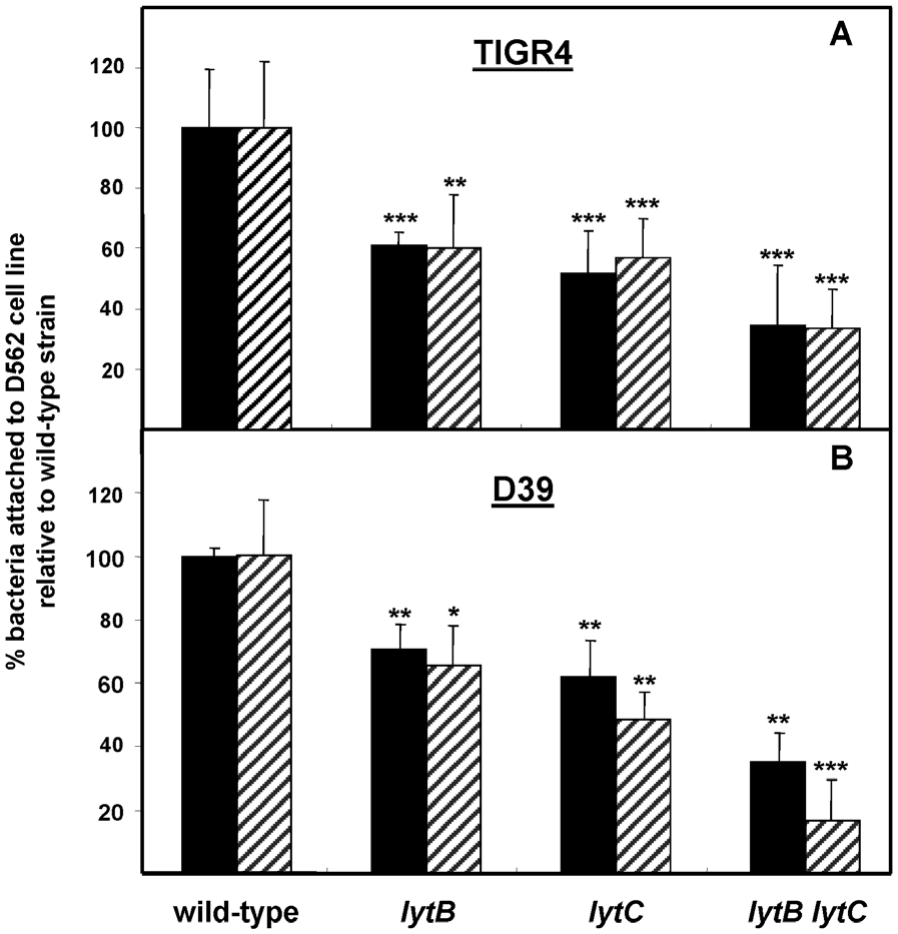
Proportion of bacteria recovered from D562 cells infected with wild-type or defective strains in CWHs. D562 cells were incubated at 37°C (solid bars) or 30°C (hatched bar). Error bars represent the SDs, and asterisks mark results that are statistically significant compared with wild-type strains (two-tailed Student's *t* test; *, *P*<0.05; **, *P*<0.01; ***, *P*<0.001). (A) TIGR4 strain and isogenic mutants. *P*<0.01 (*lytB* vs. *lytB lytC*) and *P* = 0.09 (*lytC* vs. *lytB lytC*) (Student's unpaired *t* test, 2-tailed). *P*<0.001 for the overall comparison (one-way ANOVA with a post hoc Dunnett test). (B) D39 strain and isogenic mutants. For the *lytB lytC* double mutant vs. *lytB* or *lytC* single mutants: *P*<0.001 (Student's unpaired *t* test, two-tailed). *P*<0.001 for the overall comparison (one-way ANOVA with a post hoc Dunnett test).

The ability of *lytB* and *lytC* mutants to adhere to nasopharyngeal cells at 37°C or 30°C was investigated using two different strains (TIGR4 and D39). Loss of either LytB or LytC in both serotypes caused a significant reduction in the capacity *of S. pneumoniae* to attach to the D562 cells at either temperature (Fig. 1) demonstrating that both CWHs play an important role in nasopharyngeal colonization. The proportion of bacterial attachment appeared to be slightly lower (*P* <0.05) for D39 *lytC* (48.3%±8 SD) than for D39 *lytB* mutants (65%±12 SD) when incubated at 30°C which is the optimal temperature of the LytC lysozyme activity. To investigate whether both LytB and LytC hydrolases collaborate in promoting efficient attachment to nasopharyngeal cells, double mutants were also evaluated. The proportion of *lytB lytC* TIGR4 and D39 derivatives attached to the D562 cells was greatly reduced at both temperatures compared to the wild-type and the single mutant strains, with a very significant impairment at 30°C for both serotypes (Fig. 1).

### LytB and LytC are key cell wall hydrolases in nasopharyngeal colonization

**Figure 2:**
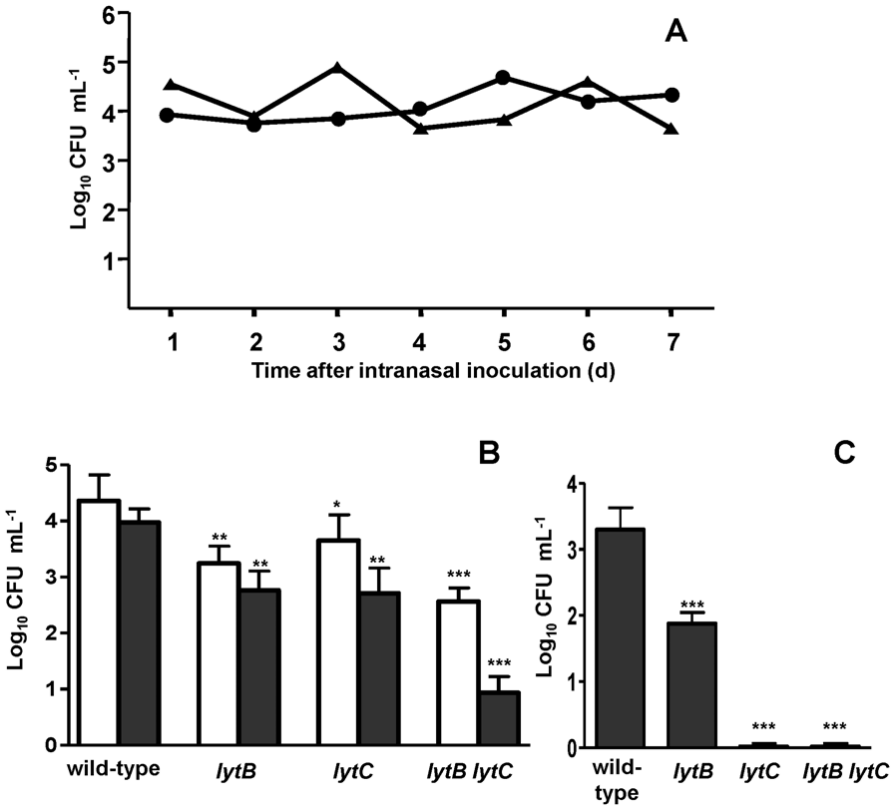
Nasopharyngeal colonization by clinical *S. pneumoniae* isolates and CWHs mutants in a mouse model. (A) Colonization curve of mice inoculated intranasally with *S. pneumoniae* TIGR4 (triangles) and D39 (circles). Number of pneumococci recovered from the nasopharynx of the infected mice is expressed as Log_10_ CFU ml^−1^. (B) Results of the nasopharyngeal colonization levels at 24 h from mice infected with TIGR4 (open bars) or D39 (solid bars) and the correspondent *lytB*, *lytC* and *lytB lytC* mutant strains. Error bars represent the SDs, and asterisks mark results that are statistically significant compared with wild-type strains (two-tailed Student's *t* test; *, *P*<0.05; **, *P*<0.01; ***, *P*<0.001). For the *lytB lytC* double mutant strain vs. *lytB* or *lytC* mutants, *P* values are: *P*<0.01 for TIGR4 and *P*<0.001 for D39 (Student's unpaired *t* test, 2-tailed). *P*<0.001 for the overall comparison (one-way ANOVA with a post hoc Dunnett test). (C) Nasopharyngeal colonization levels at 120 h from mice infected with D39 and the correspondent *lytB*, *lytC,* and *lytB lytC* mutants. Error bars represent the SDs, and asterisks mark results that are statistically significant compared with the wild-type strain (two-tailed Student's *t* test; ***, *P*<0.001). *P*<0.001 for the overall comparison (one-way ANOVA with a post hoc Dunnett test).

To investigate the involvement of LytB and LytC in nasopharyngeal colonization under in vivo conditions, we first evaluated whether a high dose of bacteria was able to induce prolonged colonization of mice. To confirm the viability in the mouse model of infection, TIGR4 and D39 wild-type strains were inoculated by the intranasal route and the concentration of bacteria present in the nasopharynx was determined. Nasopharyngeal colonization was maintained with bacterial levels between 10^4^ and 10^5^ CFU/ml (median levels 1.61×10^4^ CFU/ml and 2.51×10^4^ CFU/ml for D39 and TIGR4 strain, respectively) throughout the whole period of the study (7 days) (Fig. 2A). The animals did not develop bacteremia as blood collected daily to explore the presence of bacteria in the systemic circulation was sterile at all the time-points investigated (data not shown). *S. pneumoniae* mutants lacking either LytB or LytC showed an impaired colonizing capacity, with a dramatic effect for *lytC* along time, demonstrating that these important surface-exposed proteins are involved in the adhesion and colonization of the mucosal surfaces lining the nasopharynx (Fig. 2B–2C). Moreover, the *lytB lytC* double mutants were even less capable of colonizing the mice than the corresponding single mutants, which confirm that the combination of LytB and LytC is highly effective for the establishment of the carrier state.

### C3b deposition is enhanced on the surface of *lytC* and *lytB lytC* strains

**Figure 3:**
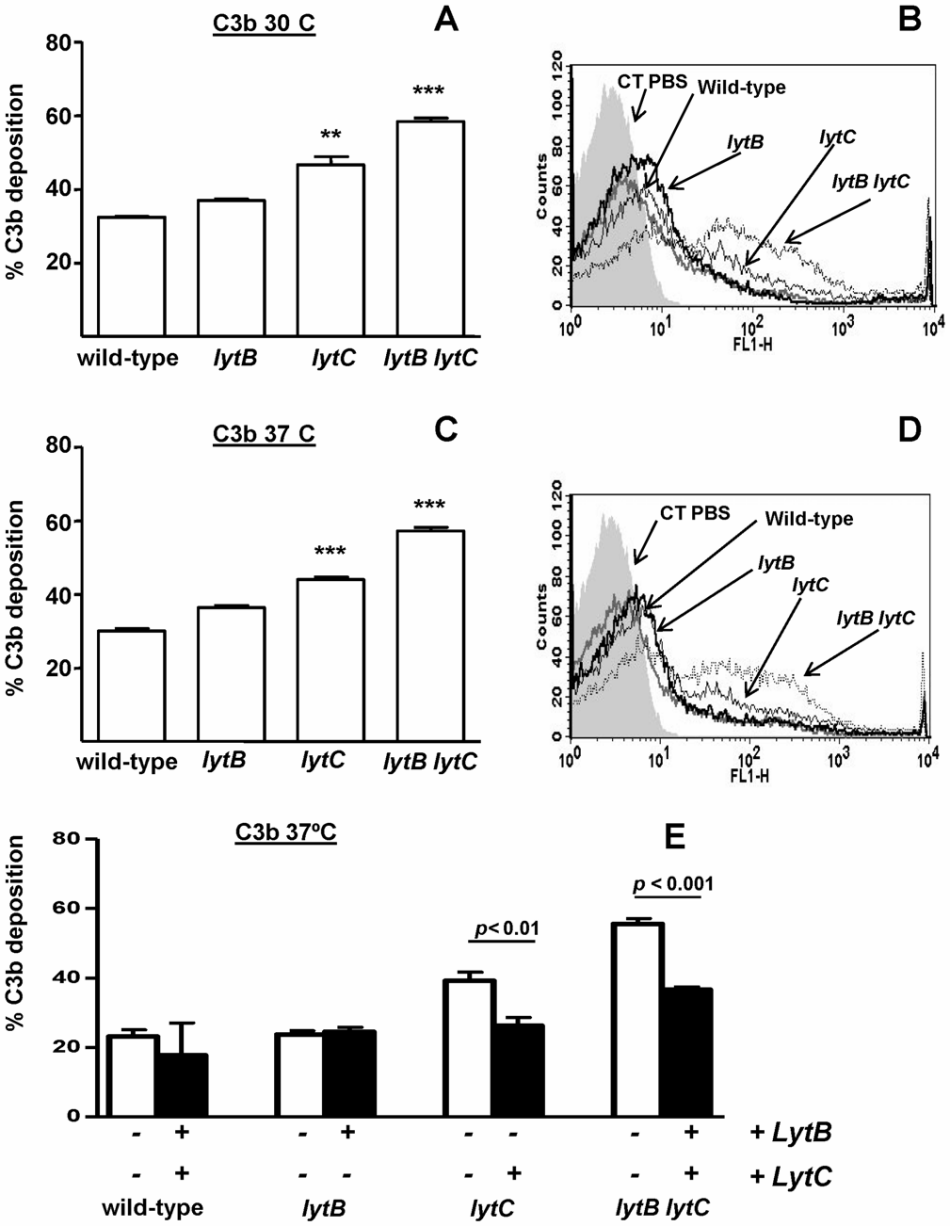
C3b deposition on *lytB*, *lytC* and *lytB lytC* D39 strains using a flow cytometry assay. (A) Proportion of C3b deposition at 30°C. (B) Example of a flow cytometry histogram for C3b deposition at 30°C. (C) Proportion of C3b deposition at 37°C. (D) Example of a flow cytometry histogram for C3b deposition at 37°C. Error bars represent the SDs and asterisks indicate statistical significance compared to the wild-type strain (two-tailed Student's *t* test; *, *P*<0.05; **, *P*<0.01; ***, *P*<0.001). *P*<0.001 (at 30°C and 37°C) for the comparison of the results for *lytB lytC* versus the single mutants. For the results for all defective strains compared to wild-type strain at both temperatures, *P*<0.001 (one-way ANOVA with Dunnett's post hoc test). (E) Restoration of the C3b levels on *lytB*, *lytC,* and *lytB lytC* mutants in the presence of 2 µg of the indicated purified CWHs. Open bars indicate C3b deposition without the addition of exogenous proteins (–), whereas blackened bars represent C3b levels in the presence of LytB, LytC or a mixture of both proteins (+).

To analyze the interaction of the different CWHs with the complement system, a flow cytometry assay was performed. C3b deposition was measured on the bacterial surface of the different mutants and the wild-type strain on a D39 background as this strain has been commonly used worldwide to investigate the interaction of different proteins of *S. pneumoniae* with the complement system [18], [28], [29]. Binding of the key complement component C3b to the *lytB* strain was similar to the wild-type strain at both 30°C and 37°C showing that LytB is not required for binding of the C3b component (Fig. 3). However, C3b deposition was increased on the *lytC* background at both temperatures suggesting that LytC avoids complement immunity by targeting C3b (Fig. 3). The double mutant was also found to bind C3b at higher level than wild-type or single mutants, confirming that the combination of both LytB and LytC is highly effective in the inhibition of the complement activity. Addition of exogenous purified proteins restored C3b levels to those found for the wild-type strain demonstrating that the increased C3b deposition on the *lytC* and *lytB lytC* mutants was due to the absence of the proteins (Fig. 3E).

### LytB and LytC hinder *S. pneumoniae* recognition and engulfment by murine alveolar macrophages

**Figure 4:**
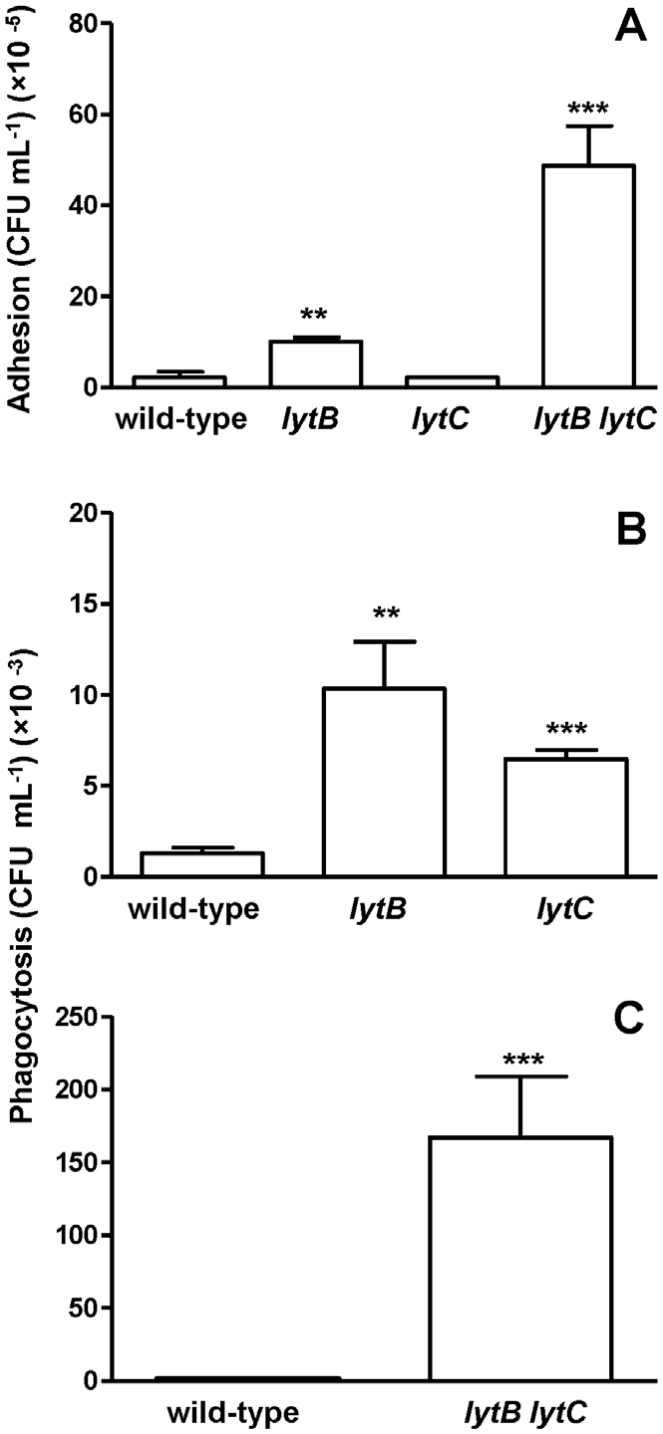
Phagocytosis mediated by murine alveolar macrophages. (A) Adhesion of D39 wild-type strain and *lytB*, *lytC* and *lytB lytC* strains to alveolar macrophages. (B and C) Phagocytosis of the wild-type D39 and CWHs defective strains by alveolar macrophages. Error bars represent the SDs and asterisks indicate statistical significance compared to the wild-type strain (two-tailed Student's *t* test; *, *P*<0.05; **, *P*<0.01; ***, *P*<0.001). *P*<0.05 and *P*<0.01 for the comparison of the results in adhesion and phagocytosis for *lytB lytC* vs. *lytB* or *lytC* respectively. *P*<0.001 for the overall comparison in phagocytosis (one-way ANOVA with a post hoc Dunnett test).

The lung contains alveolar macrophages which are both sentinels and the first line of defense against infection and clearance of *S. pneumoniae* from lungs and blood depends on the efficiency of host phagocytes to recognize and destroy the pathogen. Phagocytosis experiments were performed using the murine MH-S cell line to investigate the ability of alveolar macrophages to phagocytose the mutants lacking different CWHs compared to the D39 wild-type strain. Adhesion to the alveolar macrophage cell line was slightly, but significantly increased for the *lytB* strain compared to the wild-type strain while no difference was detected between the wild-type and the *lytC* mutant (Fig. 4A). However, phagocytosis was significantly increased for both *lytB* and *lytC* mutants suggesting that each of the CWHs are involved in evasion of phagocytosis by alveolar macrophages (Fig. 4B). Furthermore, loss of both CWHs had a marked increased on pneumococcal adhesion and phagocytosis compared to the wild-type and the single mutant strains, indicating that the presence of LytB and LytC is of great importance for resistance to phagocytosis mediated by alveolar macrophages (Fig. 4C).

### LytB and LytC enhance the resistance to neutrophil phagocytosis

**Figure 5:**
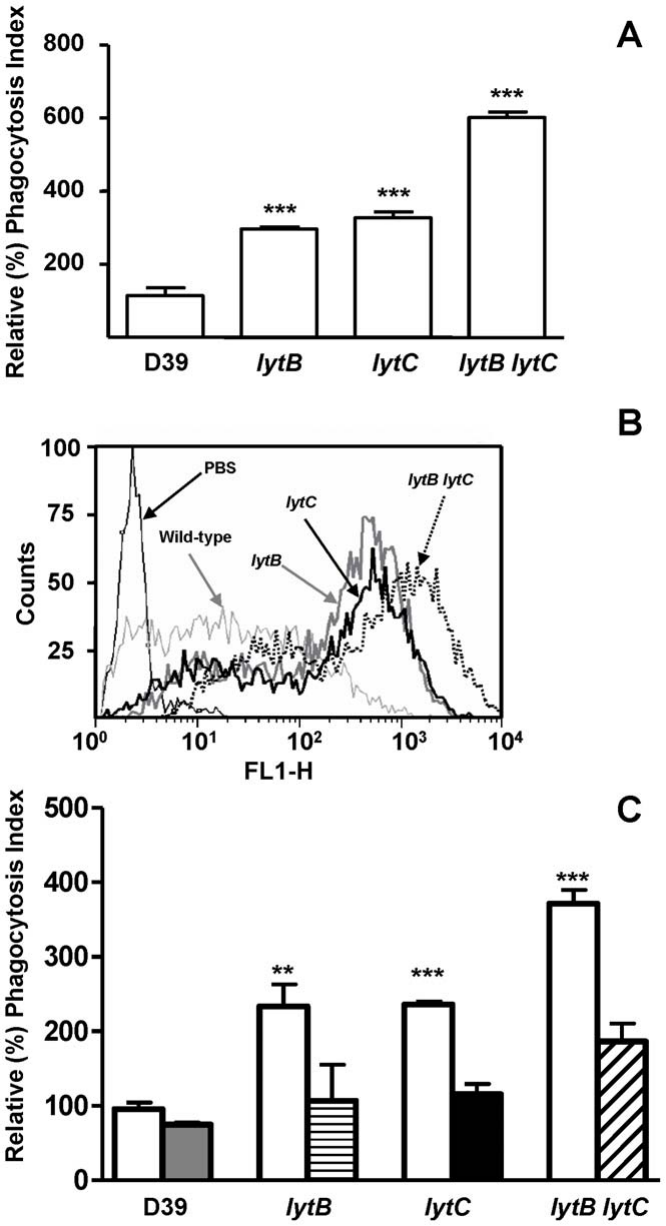
Opsonophagocytosis mediated by human neutrophils using a flow cytometry assay. (A) Phagocytosis of D39 strain and *lytB*, *lytC* and *lytB lytC* strains incubated in 20% human serum and expressed as percent fluorescent indices relative to the results for the wild-type D39 strain. (B) Example of a flow cytometry histogram for phagocytosis. (C) Restoration of the levels of phagocytosis of *lytB*, *lytC* and *lytB lytC* mutants in the presence of 2 µ]g of the indicated purified CWHs. Open bars represent phagocytosis without addition of proteins. Grey bar shows phagocytosis of wild-type strain in the presence of LytB and LytC proteins. Striped, blackened, and hatched bars indicate, respectively, phagocytosis of *lytB*, *lytC*, or *lytB lytC* mutants in the presence of LytB, LytC, or a mixture of both proteins, respectively. Error bars represent SDs and asterisks represent statistical significance compared to the wild-type strain (two-tailed Student's *t* test; *, *P*<0.05; **, *P*<0.01; ***, *P*<0.001). *P*<0.0001 for the overall comparison in phagocytosis (one-way ANOVA with a post hoc Dunnett test). For the comparison of the phagocytosis of the different mutant strains compared to the phagocytosis in the presence of exogenous proteins (*P*<0.05 for *lytB* and *P*<0.01 for *lytC* or *lytB lytC* mutants).

The complement system is a key component of the immune system involved in phagocytosis of invading pathogens in the systemic circulation and indeed, complement receptors for C3b / iC3b are one of the primary receptors on human neutrophils which mediate opsonophagocytosis of pneumococci and other encapsulated bacteria. The presence of complement receptors on HL-60 granulocytes has been previously documented [23] and therefore expression of CD11b (iC3b receptor and CR3 α-chain), a marker of granulocytic differentiation, was measured prior to phagocytic assays to confirm the presence of the receptor (data not shown). Phagocytosis of *lytB* and *lytC* single mutants was significantly increased indicating that both CWHs are involved in the evasion of uptake by human neutrophils (Figs. 5A and B). Moreover, the phagocytosis of the *lytB lytC* double mutant strain was markedly increased compared to the wild-type and the single mutants, demonstrating that LytB and LytC together, are highly effective in diverting neutrophil-mediated phagocytosis.

It is well documented that choline-binding proteins of *S. pneumoniae*, such as the CWHs studied here, bind rapidly and specifically to the choline residues located at the pneumococcal surface when added to a bacterial culture [4], [5]. To investigate further whether LytB and LytC in combination are more effective at preventing phagocytosis of *S. pneumoniae* than each protein alone, phagocytosis assays were repeated in the presence of the corresponding purified proteins. These experiments confirmed that the enhanced phagocytosis of the mutant strains was mainly due to the absence of the CWHs providing further evidence that the proteins in combination had a pronounced activity on pneumococcal avoidance of phagocytosis (Fig. 5C).

### LytB and LytC contribute to pneumococcal sepsis

**Figure 6:**
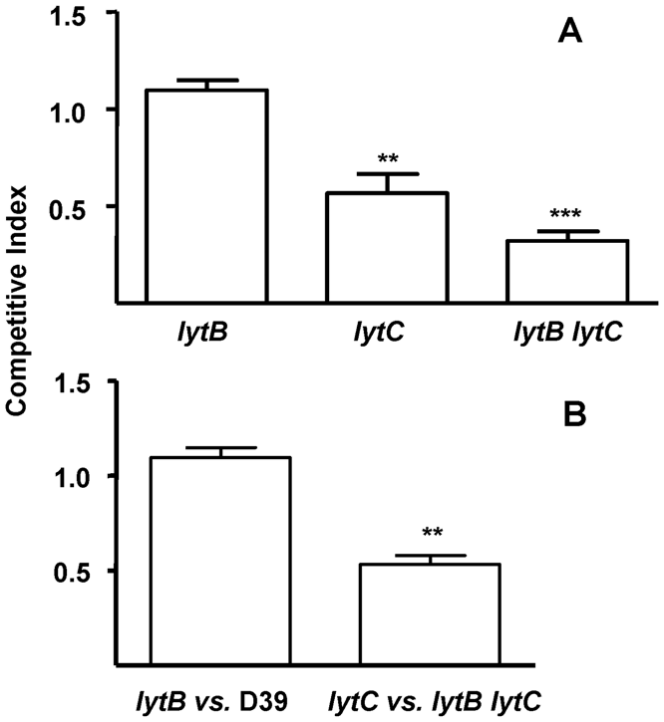
Role in virulence of the different strains using a sepsis model of infection. (A) Virulence represented as a competitive index (CI) of *lytB*, *lytC* and *lytB lytC* strains compared to their parental D39 wild-type strain. Error bars represent SDs of the means. For the comparisons of the results of the CI of the *lytC* strain vs. *lytB* **, *P*<0.01 (two-tailed Student's *t* test). For the comparisons of the double *lytB lytC* strain vs. the single *lytB* or *lytC* strains ***, *P*<0.001 and *, *P*<0.05 respectively (two-tailed Student's *t* test). (B) CIs of *lytB* strain compared to strain D39 and CIs of *lytB lytC* mutants compared to *lytC* strain to assess the combined effect of LytB and LytC in sepsis. **, *P*<0.01 for the comparison of both CIs (two-tailed Student's *t* test).

To investigate the effect of *lytB* and *lytC* mutations on the virulence of *S. pneumoniae*, CIs were determined in a mouse sepsis model. The CI for the *lytB* mutant strain was close to 1 indicating that LytB by itself does not have a significant role in the establishment of pneumococcal sepsis (Fig. 6A). However, the CI was reduced for the *lytC* mutant compared to the wild-type strain, showing a significant attenuation in virulence in this model of infection (Fig. 6A). In addition, reduced CIs were also obtained for the double *lytB lytC* mutant strain compared to the D39 parent strain, demonstrating that loss of both LytB and LytC impairs virulence significantly compared to the single mutants and the wild-type strain (Fig. 6A). To confirm that loss of both LytB and LytC results in a cooperative reduction in virulence, mixed infections of the *lytB lytC* mutant vs. the *lytC* strain were performed. As the *lytC* mutation is present in both strains, the CI (*lytC* vs. *lytB lytC*) should be similar to the corresponding CI for the *lytB* vs. wild-type strain. However, if the CI is lower, the results would indicate an additive or synergistic phenotype of mutations affecting independent functions involved in systemic infection [18], [27]. Mixed infections of the *lytC* vs. *lytB lytC* were performed in a sepsis model of infection showing a significantly lower CI than the CI found for the *lytB* strain vs. the wild-type D39 strain (Fig. 6B), confirming that loss of both LytB and LytC has additive effects on virulence.

### Lack of both LytB and LytC is associated with an impaired pneumococcal pneumonia

**Figure 7:**
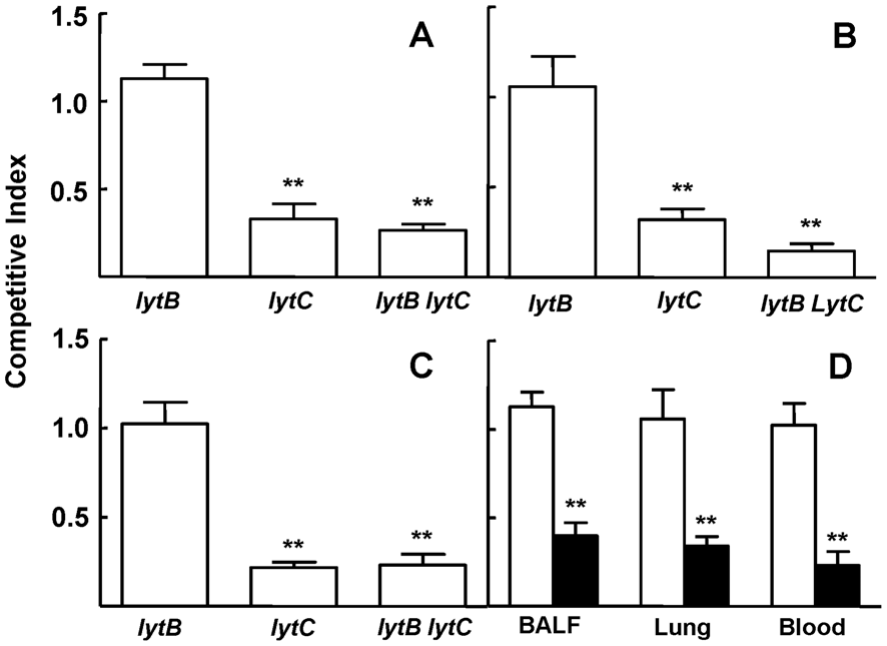
Impact of mutations in the genes encoding LytB and LytC on pneumococcal pneumonia. (A to C) CIs of *lytB*, *lytC* and the double *lytB lytC* strains compared to their parental D39 wild-type strain in BALF (A), lungs (B), and blood (C) after intranasal inoculation of a mixed culture of the corresponding mutant with the wild-type strain. Error bars represent SDs of the mean and asterisks indicate results that are statistically significant compared to those for the *lytB* strain which was outcompeted (two-tailed Student's *t* test; *, *P*<0.05; **, *P*<0.01; ***, *P*<0.001). (D) CIs of *lytB* vs. D39 (open bars) and *lytB lytC* vs. *lytC* (blackened bars) in BALF, lungs, and blood after intranasal infection. **, *P*<0.01 for the comparison of both CIs at the corresponding sites of infection (two-tailed Student's *t* test).

To further analyze the role of LytB and LytC on pneumococcal pneumonia, strains lacking each CWH and the wild-type strain were inoculated as mixed infections and their role in virulence were calculated as a CI for bacteria recovered from BALF, lung, and blood. Loss of LytB did not show impaired virulence at any of the target sites analyzed confirming that the single mutation of LytB does not have any effect on bacterial virulence within the lung airway compartment (Figs. 7A to C). The CI of *lytC* vs. the wild-type strain was significantly reduced in BALF and lung confirming that LytC is important for the establishment of pneumococcal pneumonia in the respiratory tract (Fig. 7A and B). Moreover, the bacteremia caused by the *lytC* mutant strain was reduced after intranasal inoculation compared to the wild-type strain, suggesting that LytC plays a significant role in the spread of the bacteria from the lung to the systemic circulation (Fig. 7C). The virulence of the double mutant *lytB lytC* strain was explored using competitive indices and showed that loss of both CWHs had a consistent impaired effect on virulence suggesting that the combination of LytB and LytC is of great importance for full virulence during pneumonia and invasive dissemination.

To explore whether loss of both LytB and LytC might have a cooperative role on pneumococcal pneumonia, mixed infections were performed by the intranasal route to compare the CIs of the double *lytB lytC* strain vs. *lytC* with those obtained for the mixed infection of *lytB* mutant vs. the wild-type strain. Our results show that the CI of the *lytB lytC* vs. *lytC* was between 2–3 times lower in the lung compartment (1.13 vs. 0.39 in BALF and 1.06 vs. 0.34 in the lung; *P*<0.01) and nearly five times lower in blood (1.02 vs. 0.23; *P*<0.01) than the CI of the *lytB* vs. wild-type strain (Fig. 7D) confirming that, although LytB may not have a direct role itself in virulence, this CWH improves the pathogenesis mediated by LytC in the establishment of pneumococcal pneumonia and enhances the spread of the bacteria throughout the systemic circulation.

## Discussion

*S. pneumoniae* is one of the most important human pathogens responsible for serious diseases associated to high morbidity and mortality rates worldwide [30]. Although the pneumococcal capsule is essential for full virulence by preventing complement immunity and phagocytosis [22], [31], there are strong evidences suggesting that structural components of the bacteria are also important at different steps of the pathogenesis process [32]. Identification and functional analysis of gene products involved in colonization, inflammation, and invasion is a key tool to understand the host-pathogen interaction of *S. pneumoniae* and provides essential knowledge that can be used to fight against this pathogen with the discovery of new therapeutic targets or vaccine-based strategies. Peptidoglycan is a major component of the bacterial cell envelope that can be modified by the activity of bacterial CWHs [33]. CWHs are a group of proteins whose role in pathogenesis is not fully understood, although it is thought to be important [4]. As peptidoglycan is one of the main pathogen-associated molecular patterns targeted by the host innate immune system, modifications of this essential and unique cell wall component may be used by bacterial pathogens to subvert host innate immunity [34], [35]. Peptidoglycan hydrolases are required for initial attachment to hydrophobic surfaces and contribute to bacterial pathogenesis and full virulence by increasing the survival within the systemic circulation, therefore enhancing the spread of the bacteria throughout the host [36], [37], [38], [39]. In this study we have constructed single and double mutants in LytB and LytC to investigate the impact of both proteins on pneumococcal pathogenesis by exploring their role in the establishment of the carrier state, invasive disease, and evasion of several host defence mechanisms. Our findings demonstrate that both LytB and LytC are important pneumococcal surface proteins involved in the attachment to human nasopharyngeal cells, with a more marked effect of LytC at 30°C, which is the physiological temperature found in the upper respiratory tract and the optimal temperature for this enzyme [10]. Previous results by Gosink and coworkers [14] have shown that loss of function of LytC only moderately reduced (30%) the adherence of *S. pneumoniae* to D562 cells at 30°C, whereas no effect was observed at 37°C. It should be noted, however, that those authors employed a fluorescein isothiocyanate-labeled, nonencapsulated derivative of TIGR4 strain and a very different methodology, i.e., pneumococcal cells were fixed with glutaraldehyde before counting, and adherent bacteria were quantified visually. These experimental conditions may have introduced a significant bias on the results reported by those authors. Using double *lytB lytC* defective TIGR4 and D39 strains we evaluated the role of both CWHs in adhesion to nasopharyngeal cells. Our results demonstrate for the first time that the attachment of *S. pneumoniae* to human nasopharyngeal cells was dramatically impaired in the absence of both LytB and LytC proteins with a maximum reduction at 30°C. The role of both CWHs was also investigated in a mouse model of nasopharyngeal colonization using a high bacterial challenge (10^7^ CFU). This inoculum results in colonization as well as invasion of the adjacent mucosal sites without bacteremia [40]. Bacterial levels in the nasopharynx were maintained between 10^4^ and 10^5^ CFU ml^−1^ throughout the 7 days of the study. These results confirmed that this model of nasopharyngeal colonization was self-limited and similar to pneumococcal carriage in humans [41]. Our results demonstrated that LytB and LytC are important CWHs involved in the initial attachment to the nasopharynx although other pneumococcal proteins also participate at this early stage as there was some degree of colonization in the absence of both CWHs and confirmed that the combination of LytB and LytC is required for effective colonization of the respiratory tract over time. Targeting mechanisms of preventing bacterial factors involved in the colonization process during pneumococcal pathogenesis might be an attractive strategy to prevent the pneumococcal carrier state.

Following adherence and asymptomatic colonization, *S. pneumoniae* must overcome host defense mechanisms implicated in the recognition and clearance of the microorganism to gain access to the alveolar space and/or the systemic circulation, producing invasive disease. The complement system is an important component of host immunity participating in the recognition and phagocytosis of invading pathogens [42]. Evidence suggesting that different proteins of *S. pneumoniae* are involved in the inhibition of complement immunity and phagocytosis has been previously published [18], [29], [43]. Our results demonstrate that loss of LytC, but not LytB, increased C3b deposition at both 30°C and 37°C demonstrating that LytC diverts C3b deposition on the bacterial surface. This is the first evidence showing complement evasion mediated by the pneumococcal lytic lysozyme. In addition, loss of both LytB and LytC was associated with a markedly increased C3b deposition on the bacterial surface confirming that the combination of both proteins is very efficient in the diversion of complement immunity.

The interaction with phagocytic cells was also investigated using two different cell lines that are representatives of pneumococcal phagocytosis [44], [45]. *lytB* or *lytC* single mutants displayed an increased uptake of *S. pneumoniae* by alveolar macrophages and neutrophils, whereas the phagocytosis of the double mutant *lytB lytC* was greatly enhanced compared to the single mutants. These differences do not seem to be attributable to variations in cell separation among the strains as it has been reported that the average number of pneumococci per phagocyte is the same between pneumococcal long-chain and short-chain variants [46]. Our results indicate that both CWHs are important pneumococcal proteins involved in resistance to phagocytosis and demonstrate that the presence of both LytB and LytC is highly effective in evasion of pneumococcal phagocytosis. A clear correlation exists between resistance to phagocytosis and carriage, where strains more resistant to neutrophil clearance have an advantage to persist in the nasopharynx [47]. The results of our study support this hypothesis and suggest that one of the main virulence mechanisms of LytB and LytC might be to avoid the recognition by professional phagocytes within the upper respiratory tract allowing the bacterium to efficiently colonize the nasopharynx. Targeting these proteins by the use of a vaccine or a chemical drug might help to reduce the carrier state by increasing the ability of the host immune defense system to efficiently recognize *S. pneumoniae* from the human nasopharynx. The addition of exogenous LytB and LytC proteins to the single and double mutant strains restored C3b levels to those found on the wild-type strain and increased the resistance to phagocytosis to a similar degree than in the wild-type isolate strongly suggesting that the phenotypes observed were mainly due to the lack of LytB and LytC rather than other unexpected genetic differences among the strains such as insertions, duplications or second site mutations. Moreover, the addition of choline binding proteins such as LytB and LytC to a bacterial culture has been shown to recognize specifically the choline residues located at the bacterial surface. Addition of GFP-LytB to a *lytB* mutant strain binds the specific target (the polar ends) to which it remains bound for a long time exhibiting a dispersing effect typical of LytB, whereas exogenous addition of the LytC lysozyme to a *lytC* deficient strain showed that this enzyme was kept until regulatory control by the “cured” cells [4], [5]. These observations suggest that the addition of purified hydrolases to the defective strains had the ability to compensate the phenotype due to the lack of these enzymes.

There is evidence supporting the idea that the factors needed for pneumococcal infection may also be important in nasopharyngeal colonization, as the majority of the genes required for invasive disease were found to be required for bacterial carriage [48]. The role of LytB and LytC in pneumococcal pneumonia and sepsis was investigated using a mouse model of infection. Loss of LytB did not influence virulence compared to the wild-type strain indicating that LytB by itself does not participate in the establishment of pneumonia or in systemic dissemination. These results are in agreement with those found by other authors using a sepsis model in which the virulence of a D39 *lytB* strain was very similar to the corresponding wild-type strain [49]. However, the virulence of the *lytC* mutant strain was attenuated in both models of systemic and pulmonary infection with an impaired ability to spread from the lung to the bloodstream confirming that LytC is an important virulence factor of *S. pneumoniae*. In addition, the double *lytB lytC* mutant strain was even more attenuated in virulence than the corresponding single mutants or the wild-type strain. This confirms that the presence of both LytB and LytC permits highly effective establishment of pneumococcal pneumonia and sepsis and indicates that both proteins enhance the dissemination of the bacteria from the lung compartment to the systemic circulation. This is in agreement with other authors confirming that different virulence factors of *S. pneumoniae* act in concert increasing bacterial virulence and reinforces the idea that several bacterial components of *S. pneumoniae* are needed to efficiently produce invasive disease [18], [29], [43], [50].

In summary, we have shown that LytB and LytC are surface exposed proteins that play a significant role in essential phases of the pneumococcal pathogenesis such as nasopharyngeal colonization, pneumonia, and sepsis. Our data suggest that pneumococcal cell wall hydrolases are important proteins of *S. pneumoniae* involved in the attachment of the bacteria to the nasopharynx and in the progress of the pneumococcal pneumonia and sepsis by avoiding complement immunity and phagocytosis.
